# Enhancement Technique Based on the Breast Density Level for Mammogram for Computer-Aided Diagnosis

**DOI:** 10.3390/bioengineering10020153

**Published:** 2023-01-23

**Authors:** Noor Fadzilah Razali, Iza Sazanita Isa, Siti Noraini Sulaiman, Noor Khairiah Abdul Karim, Muhammad Khusairi Osman, Zainal Hisham Che Soh

**Affiliations:** 1 Centre for Electrical Engineering Studies, Universiti Teknologi MARA, Cawangan Pulau Pinang, Permatang Pauh Campus, Bukit Mertajam 13500, Pulau Pinang, Malaysia; 2 Integrative Pharmacogenomics Institute (iPROMISE), Universiti Teknologi MARA Cawangan Selangor, Puncak Alam Campus, Puncak Alam 42300, Selangor, Malaysia; 3 Department of Biomedical Imaging, Advanced Medical and Dental Institute, Universiti Sains Malaysia Bertam, Kepala Batas 13200, Pulau Pinang, Malaysia; 4 Breast Cancer Translational Research Programme (BCTRP), Advanced Medical and Dental Institute, Universiti Sains Malaysia Bertam, Kepala Batas 13200, Pulau Pinang, Malaysia

**Keywords:** breast density, CAD, image enhancement, breast cancer, deep learning, textural

## Abstract

Mass detection in mammograms has a limited approach to the presence of a mass in overlapping denser fibroglandular breast regions. In addition, various breast density levels could decrease the learning system’s ability to extract sufficient feature descriptors and may result in lower accuracy performance. Therefore, this study is proposing a textural-based image enhancement technique named Spatial-based Breast Density Enhancement for Mass Detection (SbBDEM) to boost textural features of the overlapped mass region based on the breast density level. This approach determines the optimal exposure threshold of the images’ lower contrast limit and optimizes the parameters by selecting the best intensity factor guided by the best Blind/Reference-less Image Spatial Quality Evaluator (BRISQUE) scores separately for both dense and non-dense breast classes prior to training. Meanwhile, a modified You Only Look Once v3 (YOLOv3) architecture is employed for mass detection by specifically assigning an extra number of higher-valued anchor boxes to the shallower detection head using the enhanced image. The experimental results show that the use of SbBDEM prior to training mass detection promotes superior performance with an increase in mean Average Precision (mAP) of 17.24% improvement over the non-enhanced trained image for mass detection, mass segmentation of 94.41% accuracy, and 96% accuracy for benign and malignant mass classification. Enhancing the mammogram images based on breast density is proven to increase the overall system’s performance and can aid in an improved clinical diagnosis process.

## 1. Introduction

According to International Agency for Research on Cancer, an estimated 2.3 million new cases of breast cancer has overtaken lung cancer as the most prevalent cancer diagnosed, with cancer death rates significantly higher in transitioning nations [1]. Breast screening programs are a way to detect early signs of breast cancer and are dominated by utilizing digital mammography as the primary tool for cancer detection [2]. Additional modalities such as ultrasound are used in conjunction with mammography for denser breasts, whereas magnetic resonance imaging (MRI) is used for more progressive breast analysis for repeated and high-risk patients [3].

Breast density, as defined by the American College of Radiology (ACR), is used during clinical diagnosis that classifies the breast into four categories with increasing density: almost entirely fatty, scattered fibroglandular, heterogenous, and finally, extremely dense breast [4].

**Figure 1:**
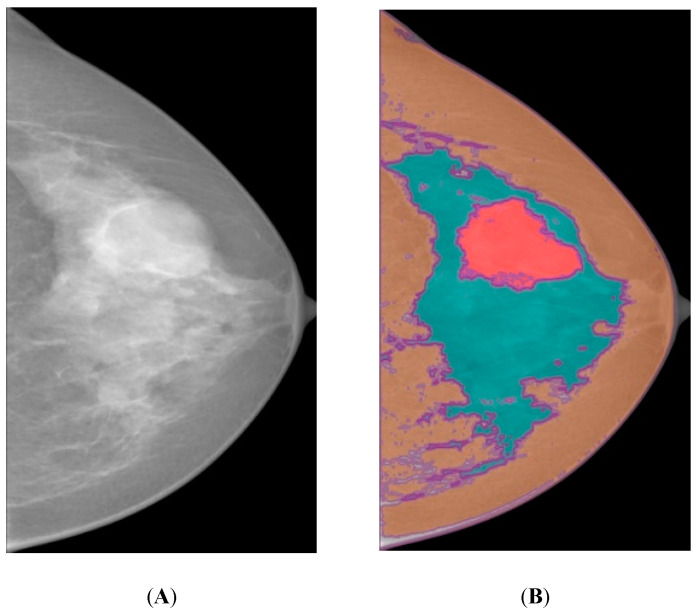
(**A**) Original mammogram image example. (**B**) Mapped tissue region for the image on (**A**)—red: mass, green: dense tissue, orange: non-dense tissue.

The heterogeneous dense breast as depicted in Figure 1A and the overlapped mass (red region) (in Figure 1B) on the dense region (blue region) is visually harder to distinguish compared to a non-dense breast that only contains mostly fatty (orange region) tissue. Diverse breast tissue structures cause mixed-intensity variations and limited visibility of breast features [5]. Due to this factor, the processed images may result in less acceptable breast tissue segmentation and inconsistent diagnosis by compromising the system’s sensitivity and specificity to detect abnormalities [6,7]. Past studies concluded that mass detection decreased with increased density, due to the mass itself being similar to the surrounding dense tissue of the breast [8,9,10]. Additionally, image quality conditions also make it difficult to detect the lesion in dense breasts [11,12]. Specifying the edge of the mass from its surrounding dense tissue requires image processing that enhances the textural element of the image as one of the defining mass descriptors to assess a mammogram visually [13]. The textural analysis identifies distinctive descriptors in the form of a changing pattern or pixel intensity with various spatial arrangements. Its refinement aims to go beyond human-eye perception by defining semantic descriptors to extract quantitative radiological data [14]. 

To accommodate the analysis of mammographic mass, Computer-Aided Diagnosis (CAD) systems are introduced to breast cancer diagnosis stages, from improving the image quality [11,15], breast lesion detection, and segmentation [16], as well as benign or malignant classification [16,17,18,19]. Moreover, CAD implementation in mammography diagnostic could reduce the human rater’s false-positive rate by 5.7% and false negative by 9.4%, as shown in a USA-based dataset [19], and an increase rate of 3% recall rate for a radiologist’s mammogram analysis with CAD assistance for an expert radiologist [20]. CAD systems proved to aid radiologists in making a better diagnosis with the area under the curve (AUC) of 0.896 from 0.850 without affecting diagnosis timing [21]. Since deep-learning CAD systems performed best when trained using large datasets [22], it is harder to apply suitable image quality improvements individually on the images, leading to a need for special enhancement procedures and careful pre-processing for the images before they can be trained on a deep-learning architecture.

Most Convolutional Neural Network (CNN) applications for CAD systems have focused on direct mammogram images for detection and classification rather than the need for specific enhancement based on breast density level and the quality of the input images. This could unintentionally lead to reduced sensitivity for mass detection in dense mammograms, resulting from higher training weightage on non-dense breasts because of dataset class imbalance [23]. Enhancement techniques based on histogram manipulation, such as adaptive/histogram equalization (HE/AHE) and contrast-limited adaptive histogram equalization (CLAHE), have been extensively used to enhance the images before training. Nevertheless, the method’s adaptability for different densities of the breast images and their effects needs to be paid attention. Several studies have included the analysis of the impact of breast density on the post-training level rather than countering its effect on the pre-processing level [10,18,24,25,26]. However, pre-processing analysis of the mass surrounded by dense tissue is essential to verify that the established CAD system is robust to dense breast images for accurate mass detection.

Based on this motivation, we proposed an enhancement technique that adapts non-dense and dense breast categories by subtly changing the non-dense region appearance within a mammogram image through textural refinement, mimicking the radiologist’s manual contrast adjustment on individual images while maintaining the visual perceptual of the original image. The textural refinement on the mass edges boosts its feature vector representability during the convolutional process for detection and segmentation algorithm for better classification performance.

In summary, this work’s contributions are focused on:

1. A breast density-based configuration is incorporated prior to the training detection algorithm.
2. An enhancement technique that enhances the textural appearance of the background and mass region by determining the threshold of the dense and non-dense region through a buffer region by manipulating the images’ lower limit cap threshold value.

## 2. Past Literature

Image enhancement is required to optimize the image’s overall quality in preparation for subsequent stages. Enhancements using histogram-based techniques have been proven to enhance mammogram images, such as through histogram equalization [26,27] and the widely used contrast-limited adaptive histogram equalization (CLAHE) [10,18,24,28,29]. Histogram-based image enhancement increases the contrast and dynamic range of the grayscale image by adjusting an image’s contrast using its histogram and increases the image’s contrast by dispersing the most common pixel intensity values by extending the image’s intensity range [30]. Researchers also combined CLAHE with their proposed method to improve their performance. For instance, CLAHE was utilized in conjunction with unsharp masking filtering, with the effectiveness in demonstrating an enhancement for mass region segmentation [31]. In addition, breast cancer detection using a modified CLAHE method is used to sharpen the margins of the masses on three datasets [32]. Meanwhile, CLAHE, wavelet, and anisotropic diffusion combination were presented for mammography enhancement in [33] and obtained a sensitivity of 93% when tested on a limited number of abnormal and normal images from the mini-Mammographic Image Analysis Society (mini-MIAS) dataset. The introduction of multilevel Otsu’s thresholding with wind-driven optimization for mass detection utilizing CLAHE enhancement on mini-MIAS and Digital Database for Screening Mammography (DDSM) mammogram datasets is conducted with 96.9% and 96.2% detection sensitivity [29]. 

Additionally, a different approach using top-hat transform-based mammography enhancement is established to increase the contrast between the suspicious area and normal breast tissues, increasing mass detection sensitivity using the proposed technique compared to unenhanced images [34]. Moreover, grayscale transformation applied by [35] helps reveal more information and increase contrast by selectively emphasizing or suppressing undesirable elements in the image, hence uniformizing the pixel distribution. Recently, a study to detect mass with its performance improved using contrast-based enhancement by employing a hyperbolic tangent function with an adjustable Tunicate swarm algorithm as optimization of the system via fitness function is demonstrated by [36] and shows improvement when compared to the CLAHE method. The use of another optimization through hybridized fast and robust fuzzy c-means clustering (FRFCM) and particle swarm optimization before mass detection was proposed on the mini-MIAS with 96.6% sensitivity [37]. A classification system for mammogram cancer by [38] using improved multi-fractal dimension features also included a pre-processing subsystem for denoising the mammogram following the cancer region segmentation.

These methods produced good final performance. However, these studies applied a straightforward object detection algorithm to analyze their method’s effectiveness for the images to be trained in a full-scale CAD system. Moreover, the enhancement methods did not take the effect of variation of breast density into consideration, with some methods causing the final mass to be indistinguishable from the dense tissue [27,31], where the final output is in the form of classification of mass and non-mass only. This could raise the issue of losing crucial mass features if continued to the cancerous mass classification stage later. The studies were also not tested against any image quality metrics as an essential aspect of any image enhancement method proposal, by using metric performance such as applied by [36], which is not considered the best in the analysis of enhancement for breast density as it relies on the contrast and intensity of the images.

Existing state-of-the-art object identification techniques such as Faster Region-based CNN (R-CNN) [39], You Only Look Once (YOLO) versions [40,41], and Single Shot MultiBox Detector (SSD) [42] have been implemented in many vision studies for detection, following the image enhancement techniques. YOLO has been proven to be the most beneficial in terms of accurate and fast detection rate [43,44] compared to the other detection algorithms. For example, mass detection using the YOLO model was carried out as proved by Al-Antari et al. [45] and resulted in a detection accuracy of 98.96%. Similarly, [28] enhanced their approach by comparing feedforward CNN, ResNet-50, and InceptionResNet-V2 for classification before implementing the YOLO model for detection. Subsequently, this team [46] proposed a CAD system framework that classified breast masses into malignant and benign using Fully Connected Neural Networks (F-CNNs). This system framework first detected breast masses using the YOLO model with an overall accuracy of 99.7%. Meanwhile, [47] employed the YOLO fusion model for breast mass detection by fusing the best feature representation from single-class mass-based and calcification-based training models to a multiclass model that combined the feature maps. Their best performance observed was 98.1% for mass lesion accuracy detection. In [48], fusion YOLO was used for detection by introducing new classes of normal and architectural distortion abnormality on final prediction with mass detection accuracy at 93% ± 0.118.

Based on the discussions, although different strategies were implemented to boost mass detection performance, the study has severe limitations that have been conducted to adapt the breast density variance effect through enhancement techniques before training the system. A fully automated mass detection based on density through CAD is crucial, especially with its link with 2.2-fold more cancer risk in clinical profiling for denser breasts reported [49]. Studies conducted by [10,18,24,25] all pointed to a decrease in the model’s performance when trained using denser breast images. One of the earliest studies of mammograms that includes adaptation to breast density developed their model using density-based spatial clustering of applications with noise (DB-SCAN), highlighting the breasts’ internal structure before training [25]. Likewise, the same method was applied by [24] on a different dataset to improve the method proposed by [25], where the author introduces a two-stage false positive reduction process through bilateral breast analysis. Even though it has good results in preparing the models based on breast density, limitations include if only unilateral breast is available, and asymmetrical factors for both breasts might affect the performance. 

## 3. Proposed Methodology

**Figure 2:**
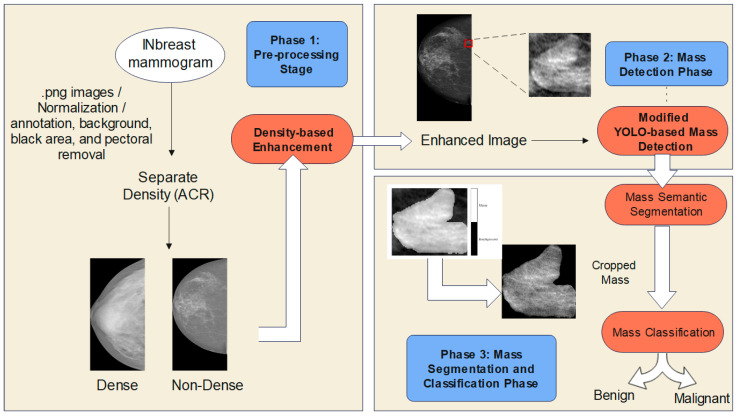
Overall Proposed Methodology for Breast Mammogram Mass Classification.

This section discusses the overall methodology for completing the framework’s three main phases, as shown in Figure 2. Each phase is discussed further in the following subsections.

### 3.1. Experimental Setting

#### 3.1.1. Dataset: INbreast

The INbreast dataset has been widely used in previous studies [18,28,50,51] and was one of the first established datasets of full-field digital mammograms (FFDM) acquired in 2011 at Centro Hospitalar de S. Joo, Breast Centre, Porto [52]. A total of 410 images were extracted with 115 abnormal lesion cases ranging from mass, calcification, and architectural distortions, with both craniocaudal (CC) and mediolateral oblique (MLO) views. Subsequently, the extracted images were exclusively updated by the authors with permission, along with the annotated ground truth range of interest (ROI) of the segmented mass region. Note that 112 mass images were included for this study that ranges across four breast density classifications, further classified based on their mass types: benign and malignant. To avoid sampling bias, 80% of the images were randomly selected for training, with the remaining 20% used for testing and validation for all stages, and were independent of the breast mass types and density level. Finally, augmentation settings were set into degrees of rotation of 30° to 300°, horizontally flipped, and scaled to randomized 1.0 to 1.3 scale factor. Augmentation settings that alter the hue, contrast, brightness, and saturation were excluded to avoid unintentional intensity changes affecting the breast density.

#### 3.1.2. Experimental Setup

This study focuses on the effect of the proposed SbBDEM enhancement technique applied in the pre-processing to prepare the images for the subsequent stages. The performance was measured by comparing the performance with the system trained using original images and two established histogram-based enhancement techniques. The final classification stage used only the handcrafted learning features to reduce the overall computation, as the mass was already accurately detected and segmented from prior stages. To compare the breast density-wise performance, the initially randomized labeled image numbering was saved from the detection phase onto the following stages to make an unbiased comparison among the same test images. Additionally, a 5-fold cross-validation was performed on the classification stage to ensure the average of using all learning features to compare performance. These experiments were visualized and executed on a workstation equipped with CPU Intel(R) Core^TM^ i7-10870H 2.3 GHz with single GPU graphic card NVIDIA GeForce RTX2060 6GB, 16 GB RAM, and trained and tested on MATLAB (Natick, MS, USA).

### 3.2. Stage 1: Proposed Image Pre-Processing for SbBDEM

#### 3.2.1. Image Preparation

**Figure 3:**
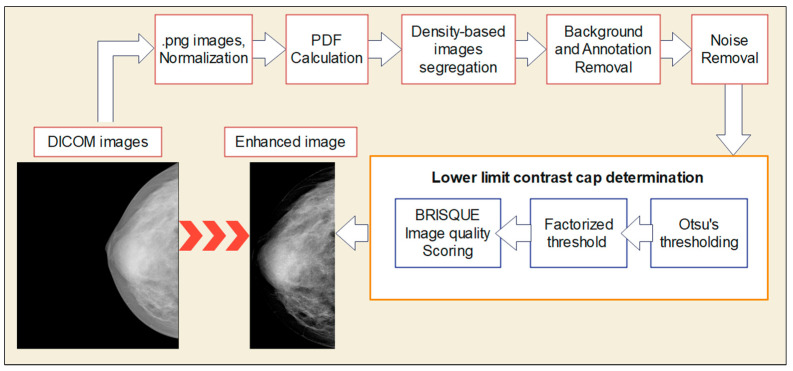
Stage 1: Proposed image SbBDEM technique as a pre-processing step.

The overall process for Stage 1 is illustrated in Figure 3. Standard morphological operations were applied to remove stray annotation marks to allow only the breast area to maximize the processing image area. To unify features between CC and MLO views, pectoral muscle was digitally removed from the MLO view images. To prepare the image to accommodate the needs of different breast densities, the images were segregated based on their supplied ACR density levels following the supplemented density scores to non-dense (1 = almost entirely fatty, 2 = scattered dense) and dense (3 = heterogeneous dense, 4 = extremely dense) categories.

#### 3.2.2. Lower Limit Contrast Cap Determination

As the next stage of the proposed framework includes mass detection process, it is essential to differentiate the mass from its background whether it is overlapped on the non-dense or dense background. To reduce the non-dense image information while enhancing features from the denser region (hence the mass), image modification was conducted by selecting the best lower-limit contrast of the image. The final output will be a breast image that have a less skin and non-dense region appearance and a pronounced textural definition of the dense region. This includes the mass region while keeping the textural features from the fibroglandular and vascular tissue of the lower-intensity fatty tissue in the background. To achieve this, the higher limit of contrast adjustment was set to the same as the original image.

#### 3.2.3. Factorized Otsu’s Thresholding for Breast Density Group Segregation

Otsu’s thresholding calculates the point value of intensity based on the image’s intensity spread on a bimodal histogram and separates the image into its foreground and background [53]. Since the original mammogram was converted to a normalized grayscale image consisting of two main tissue types that are closely related to its intensity and contrast (higher intensity = dense region, lower intensity = non-dense region), the Otsu’s value was definitive in determining the middle-intensity value that separates these tissue groups. Therefore, Otsu’s method has been implemented in this study as a reference point for determining the lower limit contrast to be clipped from the input image. However, direct Otsu’s threshold separates tissue that might belong to the other side of the histogram, such as the black background as a non-dense region and calcified vessels and the skin lining appearing white in the image as a dense region. To properly lessen this imbalance effect, the threshold value was interpolated on a scale of 1.0 to 1.9 for each non-dense and dense image group that has been separated in the previous step to subtly adapt the sudden change of region foreground to background image as a buffer intensity region. Subsequently, the training images were chosen based on their quality score, which is explained in the next stage.

#### 3.2.4. Blind/Reference-Less Image Spatial Quality Evaluator (BRISQUE)

When an image is altered, it is vital to assess it through an image quality assessment metric by referencing a gold-standard image for quality assessment in terms of its sharpness, contrast, etc., for comparison [54]. Common examples of tests where the referenced image must come from one of the images closely linked to the evaluated image include the mean-square error (MSE) and peak signal-to-noise (PSNR). However, when dealing with deep learning, possibly thousands of images are being trained, making it impossible to select only one for reference quality perspective. This is especially true if the dataset consists of multiple image acquisition techniques, which further vary the dataset’s measurement range [55,56]. In this study, to separate the overlapped mass with its background, the non-dense region becomes darker, hence enhancing the mass’s edge. This is expected to cause substantial image alteration, with mild changes on the mass and dense regions of the resulted image, causing noise to be increased in the final image. Hence, the MSE and PSNR scores are likely to produce unsatisfactory performance. Moreover, using quality assessments such as PSNR for reconstruction quality in determining the quality of an image used for a detection algorithm is unwarranted since a detection algorithm relies on its ability to separate a mass from its surroundings and, by extension, on the overall image, regardless of the final quality of the image used for training. Therefore, we chose the best Otsu’s threshold factor with an image perceptual quality evaluator known as the Blind/Reference-less Image Spatial Quality Evaluator (BRISQUE) [57]. It performed as a spatial feature image assessment metric that is commonly known as opinion-aware and analyses images with similar distortion [57], similar to how visual perception is made. As image distortions affect the quality in term of its textural features (texture signifying the difference of pixel of dense region background and the overlapped mass), BRISQUE was chosen as the primary evaluation metrics in this study. The BRISQUE score guided in choosing the optimal quality factor that clearly defines the difference between non-dense and dense breast images without using any reference image. It provides a rating by generating matching differential mean opinion score (DMOS) values using a support vector machine (SVM) regression model trained on a spatial domain image database [57]. During the training of BRISQUE, the database contained both the clean and edited versions with different additive noise implementations such as Gaussian white noise and blur, compression artifacts, and Rayleigh fast fading channel simulation, serving as the distortion image version for comparison [57]. Besides that, BRISQUE uses scenic data from locally normalized luminosity coefficients to measure any loss of naturalness due to distortion, resulting in a holistic quality score compared to calculating user-defined quality, such as ringing or blurring, as what is being measured when using PSNR [55]. Recent studies of medical images such as mammogram [58,59,60], lung CT scans [15,58], kidney and brain MRIs [15] have moved towards reference-less image quality evaluators to evaluate their work with good results. In this study, the image group was ultimately selected as the input for mass detection in the subsequent step once the best image score of BRISQUE was obtained.

#### 3.2.5. Evaluation and Analysis of the Proposed Enhancement Technique

We measured the proposed SbBDEM enhancement quality and its direct application in the input of the detection stage based on both reference-less (BRISQUE) and referenced (MSE) measurements. BRISQUE was calculated based on the method proposed by [57], and MSE was given by Equation (1):

(1) $$\mathrm{Mean}\;\mathrm{Squared}\;\mathrm{Error},\;\mathrm{MSE}=\frac{1}{mn}\sum_{0}^{m}\sum_{0}^{n}{\left|\left|f\left(i,j\right)-g\left(i,j\right)\right|\right|}^{2}$$

where *m* and *n* are the image’s height and width, *i* and *j* are elements from the enhanced image, *f*, and referenced image, *g*, whereas additional textural features analysis was made on the images based on the Gray-Level Co-occurrence Matrix (GLCM) for comparison. The texture properties extracted from the produced matrix were four statistical feature descriptors defined as contrast, correlation, energy, and homogeneity as mathematically defined in Equations (2)–(5). For every element, *P*, it reflected the total number of occurrences of the pixel values of *i* and *j* respective to the number of gray levels where σ and μ are the standard deviation and central moments derived in the form of means of variance and skewness.

(2) $$\mathrm{Contrast}=\sum_{i,j=0}^{levels-1}P_{i,j}{\left(i-j\right)}^{2},$$

(3) $$\mathrm{Correlation}=\sum_{i,j=0}^{levels-1}P_{i,j}\left[\frac{\left(i-\mu_{i}\right)\left(j-\mu_{j}\right)}{\sqrt{\left(\sigma_{i}^{2}\right)\left(\sigma_{j}^{2}\right)}}\right],$$

(4) $$\mathrm{Energy}=\sqrt{\sum_{i,j=0}^{levels-1}P_{i,j}^{2}},$$

(5) $$\mathrm{Homogeneity}=\sum_{i,j=0}^{levels-1}P_{i,j}\left|i-j\right|,$$

Additional analysis of the images’ mean intensity was evaluated for comparison. The mean intensity is the normalized mean number of normalized pixel values in each RGB channel, divided by the total number of pixels in the image, *n*, given in Equation (6).

(6) $$\mathrm{Mean}\;\mathrm{Intensity}=\frac{\sum_{n=0}^{n}\left(R+G+B\right)}{n},$$

For pixel mapping evaluation, we assessed an example of True Positive (TP) and False Positive (FP) from a sample of mass edge from the enhanced testing image using the proposed SbBDEM technique. We assessed the probability of edge detection on the next-best performed on the BRISQUE and MSE scores. Note that mass edge detection’s pixel analysis is emulated based on the first layer of modified YOLOv3 based on convolution process from Equation (7), zero padding, with a stride of two with maximum pooling downsampling to reveal the effect of pixel change made during enhancement that affects edge detection. On the other hand, diagonal edge analysis using kernel matrix *K* = [110, 10-1, 0-1-1] was chosen with a window size of 3-by-3, slides on the image using the convolution process, where *I* is the cropped mass image with *i*, *j* element, *K* represents the kernel with *x*, *y* element, and $\eta_{W},\;\eta_{h}$ and $\eta_{C}$ are the number of heights, widths, and channels of *I*, respectively. Consequently, the maximum pooling downsampled element was chosen to represent both suspected mass and background area. The edge pixel difference of Mass and Background edge detection is denoted as ∆ in Equation (8). Higher ∆ denotes the higher pixel difference between the neighboring pixel encapsulating the mass.

(7) $${\mathrm{Conv}(I,\;K)}_{x,y}=\sum_{i=1}^{\eta_{W}}\sum_{j=1}^{\eta_{W}}\sum_{k=1}^{\eta_{C}}K_{i,j,k}I_{x+i-1,y+j-1,k}$$

(8) $$\mathrm{Edge}\;\mathrm{pixel}\;\mathrm{difference},\;∆\;=\;{\mathrm{Max}}_{\mathrm{conv}}(\mathrm{mass})\;-\;{\mathrm{Max}}_{\mathrm{conv}}(\mathrm{background})$$

### 3.3. Stage 2: Mass Detection Using Modified YOLOv3

#### 3.3.1. You Only Look Once (YOLO)

Object detection is a process of detecting a specifically trained object within an image. YOLO and its versions (v2, v3, and so on) implement a single forward-pass filter by splitting the original image into a grid of s-by-s size. Subsequently, a bounding box prediction will be made for each separated cell. The algorithm searches for the object’s midpoint during training, where the specific cells containing the midpoint will be responsible for determining the target object’s presence. The corresponding cells are linked to the cell with the midpoint, which is set up as the cell with the midpoints defined as the bounding box, which is made of four components [*x*, *y*, *w*, *h*]. Here, *x* and *y* are the top left-most coordinates of the bounding box with a value of 0 to 1.0, while *w* and *h* are the width and height of the box, respectively. Both *w* and *h* could be greater than 1.0 if the final detected box is wider than an entire s-by-s cell. In addition to the four components, each box has a probability value that indicates the presence of an object in the cell and the number of class predictions. Based on this prediction value, the trained network for each cell should be able to output a specific box coordinate that contains the highest probability value for the final detected output for class prediction.

#### 3.3.2. YOLOv3 Modification for Mass Detection

This study utilized the simplest form of YOLOv3 using SqueezeNet [61] as its base network and modified it to improve the overall detection result. Note that the SqueezeNet has only 1.2 million learnable parameters as opposed to the original DarkNet-53 [40] network, which has 41.6 million parameters. As a result, SqueezeNet-based YOLOv3 was chosen to lessen the burden of weightage parameter training. Among the benefits of using a simpler network architecture are more efficiently distributed training parameters, more use of spatial information, which leads to shorter training times, less bandwidth for future model updates, and the ability to be deployed with less memory configuration [62]. Aside from being lightweight, using predefined anchors and detection heads introduced in YOLOv3 architecture allows smaller objects to be detected [40]. Depending on the base network, the YOLOv3 could extract deep features to extract three-scale feature maps from the anchors used for the final bounding-box calculation to predict the best confidence score (CS). YOLOv3 has also been successfully implemented in recent mammogram studies [63,64], showing that its implementation is reliable with good results. A comparison of YOLOv3 and YOLOv4 conducted by [65] shows that even though YOLOv4 is an improvement, it shows no substantial difference between the two models, leading the author to infer that the performance of YOLO primarily depends on the features of the dataset and the representativity of the training images.

**Figure 4:**
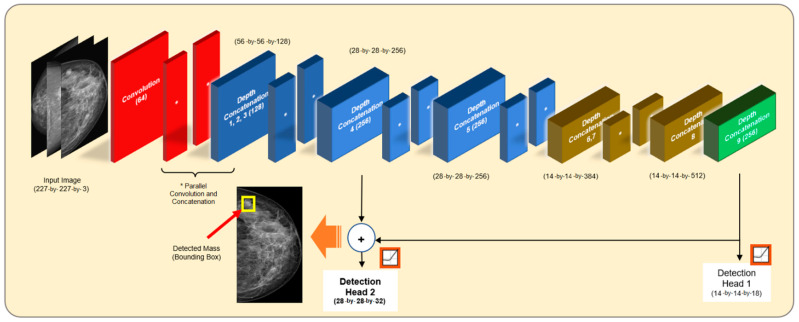
Modified SqueezeNet CNN architecture used for YOLOv3 training. The modified layer is in the Bold setting.

Figure 4 illustrates the modified SqueezeNet CNN architecture for the mass detection stage in this study. The input image size was set to 227-by-227, where the enhanced input training images were trained with whole mammogram images. The image went through a series of cascaded and parallel convolutions with concatenation along the nine repeated layers, reducing the information and computation by compacting feature maps as the network went deeper. Two detection heads were allocated when this architecture was modified for detection purposes in YOLOv3. The second detection head was double the size of the downsampled input (28-by-28) of the first detection head (14-by-14), causing smaller masses to be better detected. Since the mass size ranged from the aspect ratio of the breast size, with more than 50% of the training data containing mass with a size less than a sixth of the overall images, we have tried to resolve this problem by devising this architecture by modifying the input of the second detection head.

Hence, to improve the detection of small masses and overall detection performance, we proposed two strategies to solve this problem.

Strategy One: Residual feature mapping for the second detection head: Features from the shallower layer were included (depth concatenation four), containing higher spatial features from the skip connection, and were elementwise added with the semantic features from the deeper layer (depth concatenation nine), where the element-wise addition reduced feature degradation that occurred during downsampling which enhanced feature contrast and feature discrimination [51]. 

Strategy Two: An additional anchor box assigned to a smaller feature map: This anchor box was introduced to the lower scale of the anchor box number of the second detection head (ratio of 4:3 to first detection head). While simply increasing the number of anchor boxes increased the predefined mean intersection over union (IoU), this could only lead to lower performance due to overfitting the number of bounding boxes per image mapping [66]. However, assigning an extra anchor box only for the smaller feature map specifically will increase the bounding box refinement on the feature map allocated to features coming from Strategy One, which increases the possibility of detecting smaller mass sizes coming from the images’ semantic information.

The image gave seven predictions with their confidence level scores on every single grid cell with the size of s-by-s. The network was trained on 80 epochs with 10 mini-batch sizes. The learnable parameters were updated through a loop of stochastic gradient descent momentum (sgdm) solver. The initial learning rate was set to 0.001, and a 0.5 confidence score (CS) threshold value was defined for determining the overall mean Average Precision (mAP) score for mass detection, with the largest CS bounding box score selected for final prediction. It is important to note that the hyper-parameter tuning values were chosen based on previous studies and this study’s repeated trial processes [67]. 

#### 3.3.3. Performance Evaluation of the Modified YOLOv3 Using Enhanced Images

In this study, mass detection performance was correlated with the image enhancement performance in the prior stage. Therefore, we assessed TP and FP, while the mAP was calculated from the area under the curve of recall and precision, following Equation (9):

(9) $$\mathrm{mean}\;\mathrm{Average}\;\mathrm{Precision},\;\mathrm{mAP}=\frac{1}{\left|classes\right|}\sum_{c\in classes}\frac{\left|TP_{c}\right|}{\left|FP_{c}\left|+\right|TP_{c}\right|}$$

where *c* is the number of classes. The mAP is the current metric used by computer vision researchers to evaluate the robustness of object identification models. It incorporates the trade-off between precision and recall, which optimizes the influence of both metrics, given that precision measures the prediction accuracy and recall measures the total number of predictions concerning the ground truth.

### 3.4. Stage 3: Mass Segmentation, Feature Extraction, and Classification

#### 3.4.1. Mass Segmentation and Evaluation

Following Stage 2, the final evaluation of the system’s performance was based on its mass segmentation and classification. To fully separate the mass from its surrounding tissue, we utilized deep-learning-based semantic segmentation once the mass had been localized using the bounding box location obtained from the previous stage. Here, the highest CS was selected for more than one detection. Clearly, segmented mass is important in defining the area in which the features are extracted from the images when classifying the mass into benign or malignant in later stages. Therefore, the evaluation for segmentation performance from the Jaccard index, *J*, of the IoU score was calculated based on Equation (10):

J(A, B) or Intersection over Union, IoU = |A ⋂ B|/|A ⋃ B|(10)

where A is the sample data being tested against sample data B (ground truth sample). A higher *J* or IoU score brings better similarity between the two sets. The accuracy of the segmentation was measured based on its testing performance on different input image settings, based on Equation (11), utilizing TP, FP, TN, and FN.

Accuracy, Acc = (TP + TN)/(TP + FN + TN + FP)(11)

#### 3.4.2. Feature Extraction

In the final stage, the segmented mass was used to classify whether the mass is benign or malignant. Furthermore, handcrafted features were used to finally classify the mass into benign or malignant using a well-known machine learning technique. In this study, textural features were chosen as the main feature contributor. The segmented mass features were extracted based on three primarily used radiomics handcrafted features for mammography: textural feature (Gray-Level Co-occurrence Matrix (GLCM)), geometrical feature (mass circularity), and first-order statistics (mean intensity).

##### Feature Extraction: Gray-Level Co-Occurrence Matrix (GLCM)

The GLCM can highlight specific properties of the spatial distribution of the gray levels in the texture image. The proposed SbBDEM procedure was applied to increase the textural refinement of the dense and mass region in the earlier stage. Since both benign and malignant region segmented does not change in respect of illuminance when exposed to light, textural analysis is also essential in extracting important features to differentiate between two neighboring pixels [68]. The features were calculated based on Equations (2)–(5) as previously discussed in Section 3.2.5.

##### Feature Extraction: Circularity and Mean Intensity

A malignant breast mass varies in that its edges are uneven and likely to expand quicker, giving it a projecting look in a mammogram. In contrast, a benign mass differs because its geometric limits are more clearly defined, smooth, and consistently formed [26]. These are some of the features selected by radiologists when making visual clinical mammogram evaluations. As a result, one of the descriptors used in previous studies [25,26] is the mass’s circularity characteristic, determined using Equation (12), that is implemented using the segmented region’s area and perimeter.

(12) $$\mathrm{Circularity}=\frac{4\left(Area\right)\left(\pi \right)}{Perimeter^{2}}$$

Additionally, the inclusion of the supplementary characteristic of the mass’s mean intensity is based on the notion that since malignant mass cells are more densely formed than benign mass, it may appear to have a greater overall image intensity. The features were calculated based on Equation (6).

#### 3.4.3. Mass Classification and Evaluation

All the features were trained with and without any feature selection or reduction method using a supervised weighted *k*-nearest neighbor (*k*-NN) algorithm [69,70]. To determine the proper *k* for the training images, we ran the *k*-NN algorithm with different values of *k* and chose the *k* that minimizes errors while preserving the system’s capability to make accurate predictions when given new testing data. To make an unbiased test performance of the features, 5-fold cross-validation was applied during training, with the final *k*-neighbors value set to 10, using Euclidean distance measurement, having inverse distance weighting for the multivariate interpolation of the data points applied.

The mass abnormality classification’s performance was based on the testing accuracy as in Equation (11) and the area under the Receiver Operating Characteristic (ROC) curve. The ROC curve is a standard measuring the degree of separability of binary classification between the mass and its background on a plot of sensitivity (TP Rate) against the specificity (FN Rate), where the highest area under the ROC curve represents the model’s ability to segregate the class better.

## 4. Results and Discussion

In this section, the results are discussed based on the stages of experimental procedures explained in the previous section. Comparison of the result of the proposed SbBDEM technique in the pre-processing stage is made based on the performance of the immediate stage of mass detection and is compared between original, adaptive histogram equalization (HE/AHE), contrast limited adaptive histogram equalization (CLAHE), and the proposed SbBDEM technique in this study on all mammogram images.

### 4.1. Image Quality and Textural Elements

**Figure 5:**
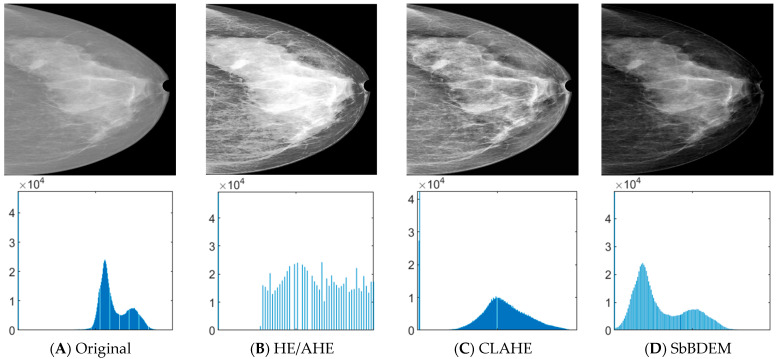
Sample images and histogram plots from columns of (**A**) Original, (**B**) HE/AHE, (**C**) CLAHE and (**D**) SbBDEM image enhancement techniques for comparison.

The performance of the proposed image enhancement in the pre-processing stage before mass detection was investigated based on differently trained image input for the models. Figure 5 shows an example of mammogram and its respective histogram for comparison on the (A) original, (B) HE/AHE, (C) CLAHE, and (D) proposed SbBDEM techniques images. Comparison of histogram for the original in Figure 5A shows similar shape to the proposed SbBDEM in Figure 5D, however its pixel distribution has expanded and shifted to the left side of the histogram. This suggested that the proposed SbBDEM can retain the pixel distribution as similar as possible to the original image, but with the decrease of intensity resulted to increasing the pixel belonging to the non-dense region. More pixels of <0.5 are extrapolated causing non-dense area to be darkened, leaving the dense and mass area lighter for better edge difference for the network to learn. 

Meanwhile, Table 1 shows the average scores for mean-square error (MSE), Blind/Reference-less Image Spatial Quality Evaluator (BRISQUE), image intensity, and GLCM statistical features comparison between the proposed SbBDEM against other enhancement techniques for all mammogram images. The BRISQUE score is improved from 43.5799 in the original image to 42.3841 and the lowest amongst others, suggesting that using the proposed SbBDEM produced an acceptable quality image in terms of better perceptual ability. Additionally, the average correlation feature for the proposed SbBDEM is the lowest at 0.9752. Since correlation measures how correlated a pixel is to its neighbor over the whole image, it is easy to conclude that neighboring pixels within the proposed SbBDEM image correlate the least with each other. This supports the better edge difference between the pixels within the image for better textural perception. Meanwhile, the energy property represents the estimated pixel attribute energy values that make up an image’s energy properties [71,72]. The energy features combine to create an image weight model, which is a collection of weights reflecting the importance of the image pixels from the perspective of perception. The higher energy property in the proposed SbBDEM image suggests the overall pixel carrying more weight is expected to be represented during network training. Finally, the contrast and homogeneity properties show no reflection to the proposed SbBDEM technique as neither shows the least or the most out scores to form varying spatial pattern arrangements.

**Figure 6:**
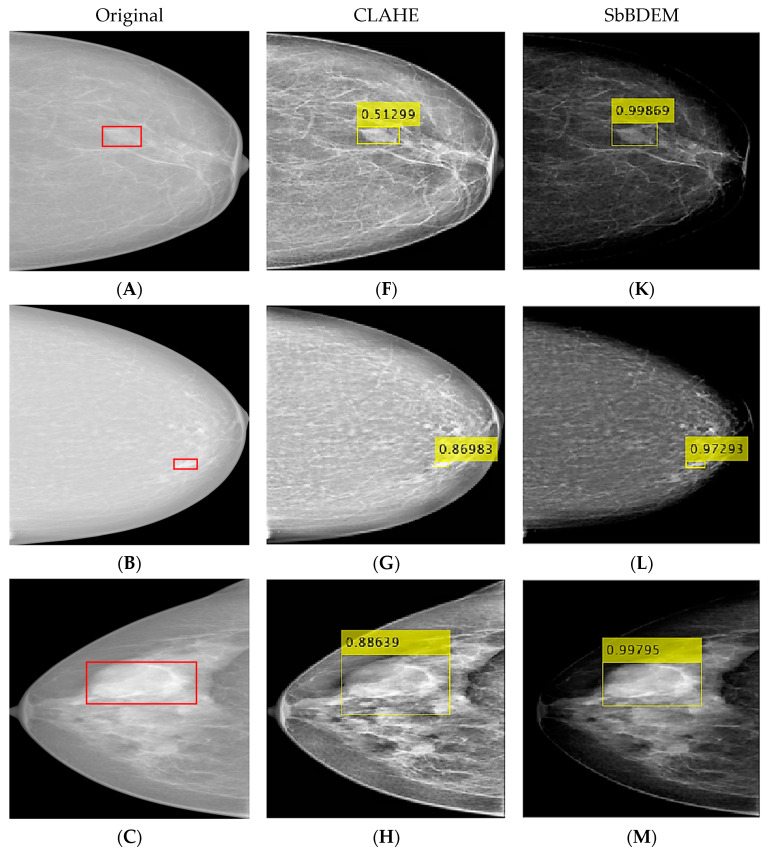

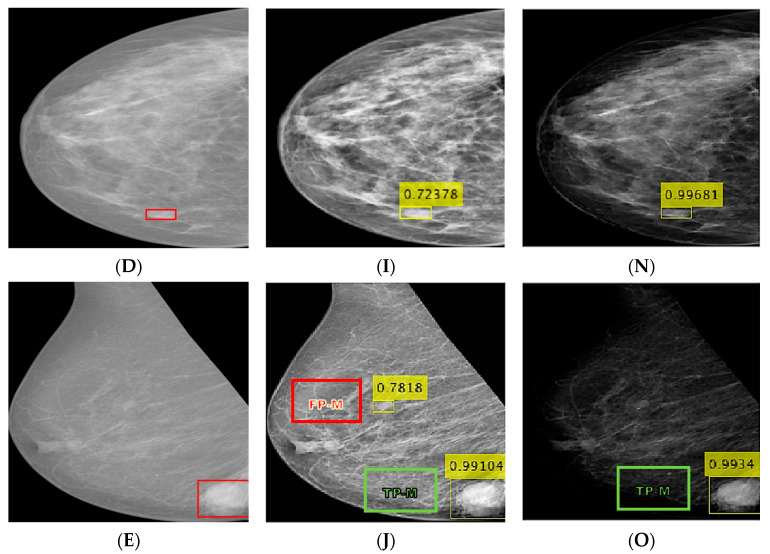
Result of Mass Detection for comparison. Rows 1 and 2: non-dense breasts. Rows 3 and 4: dense breasts. Row 5: Example of image with True Positive mass (**TP-M**) and False Positive mass (**FP-M**) detections. Yellow boxes indicate bounding boxes with a confidence score for mass detection. (**A**–**E**): Original images. (**F**–**J**): CLAHE-enhanced images. (**K**–**O**): proposed SbBDEM images.

For breast mass analysis, the result from the CLAHE-enhanced image, the enhancement technique used in most past studies [10,18,24,45,51,68] is selected to be compared to the proposed SbBDEM method. Figure 6 illustrates sample images from the result of mass detection for both non-dense (Rows 1 and 2) and dense (Rows 3 and 4) images with the confidence score (CS) indicated in the yellow boxes obtained from the mass detection stage in this study. Here, the original image on the first column Figure 6A–E with the ground-truth labeled in red boxes is followed by its respective CLAHE-enhanced (second column) and the proposed SbBDEM technique (third column) images.

Visual evaluation of the images demonstrates increased and interpolated contrast stretching observed on the CLAHE-enhanced image in Figure 6F–J. Meanwhile, the proposed SbBDEM images produced darker overall contrast, as seen in Figure 6K–O, especially on the non-dense fatty tissue region, while preserving the mass and dense region intensity from the original image. Maintaining the pixel information of the mass is essential in feature extraction and convolution of the YOLOv3 algorithm, as this will also preserve the edge of the mass during enhancement.

**Figure 7:**
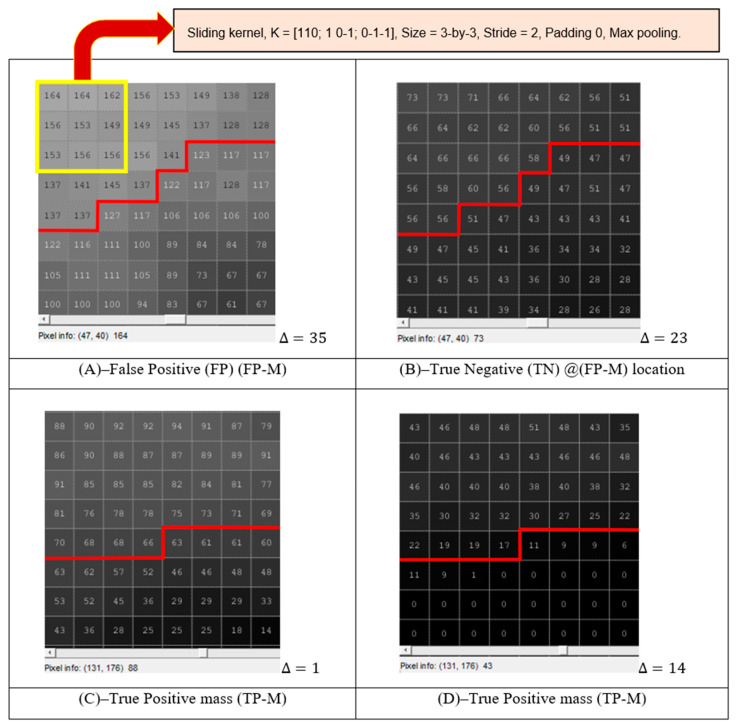
(**A**) FP detected mass edge on CLAHE image. (**B**) Corresponding location of TN mass location based on (**A**) on the proposed SbBDEM image result. (**C**) TP detected mass edge on the CLAHE image. (**D**) Corresponding TP location of detected mass location based on (**C**) on the proposed SbBDEM image result. The analysis is made from Figure 6E,J,O, where ∆ is the pixel edge difference. The lighter region above the red lines indicates the mass region.

Other than that, Row 5 of Figure 6E,J,O demonstrates an example of True-Positive Mass (TP) (TP-M) and False-Positive Mass (FP) (FP-M) detections during the mass detection stage. Further pixel analysis based on edge detection emulated by the network’s convolutional process is extracted using an 8-by-8 grid window size on the edge of expected mass FP-M corresponding to Figure 7A,B, and mass TP-M in Figure 7C,D.

The mass edge analysis is based on the difference of maximum pixel ∆ in the region where the region above the red line is the ground-truth-based mass, while the opposite is the background based on the convolution filtering process using kernel K = [110; 1 0-1; 0-1-1] and maximum pooling (Max pooling) downsampling. This revealed that the FP-M detected in Figure 7A on the CLAHE image has a higher probability of being detected based on its pixel region difference, ∆ = 35 compared to ∆ = 23 on the same pixel location on the proposed SbBDEM image in Figure 7B, as per the ground-truth in Figure 6E. Additionally, TP-M was detected on the CLAHE image and the proposed SbBDEM image. However, even though the proposed SbBDEM image is visually darker, the TP-M detected in Figure 7D for the proposed SbBDEM has a far higher mass edge detection difference at ∆ = 14 compared to its counterpart in Figure 7C using CLAHE enhancement, having ∆ = 1. This indicates that the new intensity value replacing the original pixel during the proposed SbBDEM process lowers FP detection on non-mass locations, as high-level spatial image features such as edge and coarse textures are extracted at the earliest learnable layer during YOLOv3 learning. At the same time, it increased the probability of detecting TP mass on the proposed SbBDEM image.

**Figure 8:**
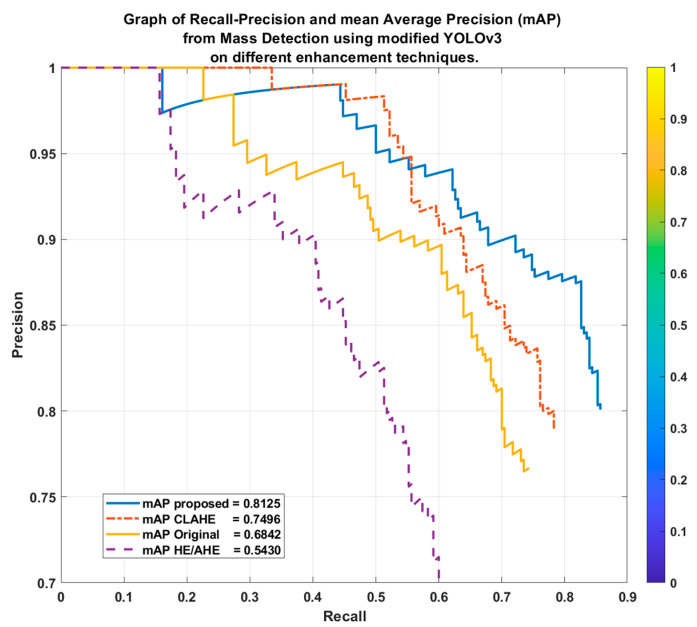
Graph of Recall-Precision Curves and mean Average Precision (mAP) from Mass Detection using modified YOLOv3 on different enhancement techniques.

The mass detection performance of the overall image enhancement is made through the next stage. It is explained from the Recall-Precision Curves (RPC) in Figure 8 for models trained with the original, HE/AHE, CLAHE, and the proposed SbBDEM images. High recall and high precision are both represented as high areas under the RPC, where high precision is correlated with a low false-positive rate, and high recall is correlated with a low false-negative rate. Note that the proposed SbBDEM enhancement technique produced the highest mean Average Precision (mAP) as area under the RPC of 0.8125, followed by CLAHE images with mAP = 0.7496. In contrast, the HE/AHE images downgraded the performance from using the original images, with mAP at 0.5430 compared to 0.6842 for the original images. This result shows that refining the textural of the mass of the original pixel that further apart the difference between the mass and its neighboring non-dense or dense region background is important to preserve its edge without diminishing the mass itself. The result also justifies that improving the images based on breast density before extracting training features is essential to increase the final overall detection performance.

**Figure 9:**
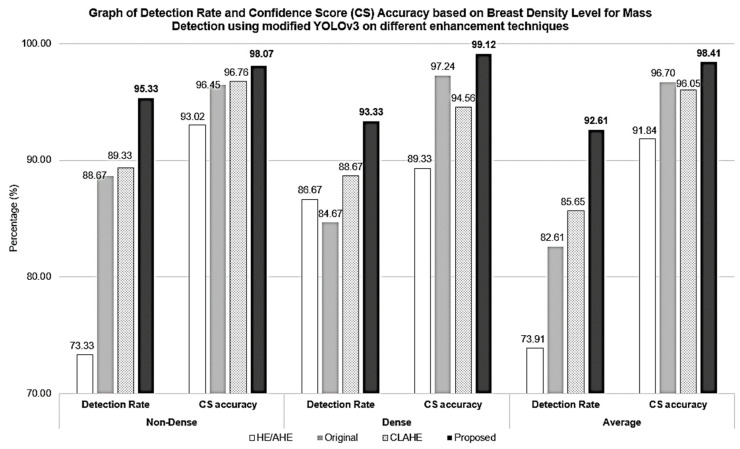
Graph of Detection Rate and Confidence Score (CS) Accuracy based on Breast Density Level for Mass Detection using modified YOLOv3 on different enhancement techniques.

Figure 9 presents a bar chart showing the comparison of performance between dense and non-dense breasts for mass detection on different image enhancement techniques. On average, the ability of the model to detect mass per image is shown on the overall performance showing the best mass detection when using the proposed SbBDEM images, followed by CLAHE, the original images, and finally, HE/AHE shows lesser performance compared to the original images. The lesser HE/AHE performance is in conformance with previous research [25] where HE/AHE might benefit in its application on RGB to HSV images in terms of gamma correction. Therefore, it is somewhat unsuitable in a grey-level image such as a mammogram, as it can only raise the contrast of the background noise while simultaneously reducing the amount of signal that can be utilized.

As for CLAHE, although it improves mass rate detection by ±3%, the overall CS shows slightly lower performance than in the original image. Compared to other techniques, CLAHE operates on tiles rather than the overall image, in which the tiles are enhanced individually, resulting in a locally stretched contrast masking on the homogeneous areas that are limited to avoid amplifying any noise that might be present in the image [68]. This might contribute to the effect of introducing FP cases on the unrelated dense region within the image that was enhanced, giving a similar feature pattern to the mass. Meanwhile, an improvement of 10% from the original image for detection rate and a slight improvement of 2% for CS accuracy is observed when the proposed SbBDEM technique is applied for mass detection. This supports the reason that contributed to its higher performance is its ability to retain the mass and the denser region as it is while reducing the non-dense region pixel value in the background. In return, a prominent spatial feature defining a mass, such as its edge, is enhanced and contributed to the feature mapping extracted in the YOLO layers, resulting to better detection rate and CS accuracy.

On average, the detection rate of the proposed SbBDEM improved to 92.61% using the proposed SbBDEM technique, followed by CLAHE, original, and HE/AHE at 85.65%, 82.61%, and 73.91%, respectively. By standardizing all test images to only the detected images for all enhancement techniques, the CS accuracy, which measures the bounding box accuracy, is highest on average when the model is trained using the proposed SbBDEM with 98.41% accuracy. Nevertheless, CLAHE-enhanced images have a lower CS accuracy performance than the original image, which may be caused by additional FP detections where the overlapping bounding box may contribute to a wider range of overlapping intersections shared on the same image, resulting in a lower CS accuracy score.

On the other hand, non-dense breast exhibits better performance compared to dense breast, as supported by previous studies [10,18,25] on all enhancement techniques for both detection rate and CS. The highest CS accuracy using the proposed SbBDEM method is at 98.07%, showing a boost of 1.62% in performance from the original image for non-dense breast and increase of 9.79% of CS for dense breast. Even though the detection rate for dense breasts is slightly lower at 93.33% than non-dense breasts at 95.33%, the CS accuracy is observed to be slightly better at 99.12% in the dense breast than in non-dense breasts at 98.07%. Additionally, note that the dense breast detection rate improvement is the best, with an increase of 8.66% from the original image. The analysis of mass detection on the denser background proves that by using the proposed SbBDEM technique, the overlapped mass detection could be improved.

### 4.2. Analysis of Modified YOLOv3 Performance

In this study, a modified convolutional neural network (CNN) for YOLOv3 is developed to evaluate the input images. Furthermore, the modification is utilized to detect the mass’s location in the mammograms by improving its ability to receive spatial features enhanced from the proposed SbBDEM technique. Table 2 presents the result of mAP performance for mass detection on the original and other enhancement image input settings with and without YOLOv3 modification for comparison.

The result displays a pattern of increasing detection performance for all image input settings on the modified YOLOv3 model, except the HE/AHE enhancement input image. The highest mAP of 81.25% is observed using the proposed SbBDEM on the modified model, with an increase in performance of 17.25% compared to using the original image on the non-modified YOLOv3 model. In this study, the modification is crafted to focus on the use of spatial features retained from the proposed SbBDEM training images. Its textural features have been improved based on the result observed from using the proposed SbBDEM technique discussed previously in Table 1. This textural refinement is further taken advantage of as an essential higher-level spatial feature extracted during training by adding the features from the earlier YOLOv3 layer to the second detection head specifically used to detect a smaller object from its initial development setting [40]. Moreover, the extra larger anchor box value that is assigned to these features gives extra weightage and encapsulates the detected mass region through the overlapping of bounding box tiled across the image, further improving the detection performance, resulting in better intersection over union (IoU) placement, given the multi-sizes of the mass on the input images [49].

### 4.3. Performance of Mass Segmentation and Classification

After localizing the position of the mass on the image, the mass region is segmented for the ease of feature extraction for classification in this study. Table 3 compares segmentation results by applying the proposed SbBDEM against the original HE/AHE and CLAHE enhancement techniques. A slight improvement in segmentation accuracy can be observed when using the proposed SbBDEM technique by achieving a mean accuracy of 0.9437 from 0.9431 from the original image. Since the mass is well contained within the bounding-box, less overlapping of mass and dense background issue needs to be resolved using the proposed SbBDEM technique. Nevertheless, the proposed SbBDEM technique also produces the highest accuracy along with IoU for both classes of mass and its background.

Meanwhile, we employed handcrafted features from the segmented mass region with and without using the principal component analysis (PCA) feature reduction method for benign and malignant classification. Comparison is also made using the chi-square-based feature selection method by removing features having a chi-square score of less than 1.0 as correlated features during training. The result shows the highest testing accuracy for benign vs. malignant mass of 96.0% is achieved on the training time at 0.670 s.

Additionally, a comparison of mass detection results of the past studies and similar methods are listed in Table 4, with and without breast density consideration before or after analysis performance, as well as the computational cost for each algorithm’s deployment. In this study, the main objective is to validate the performance of object detection utilizing the simplest CNN of SqueezeNet for a modified YOLOv3 using a differently enhanced input image, specifically to improve the performance for the detection of mass in dense breast mammograms. Similar works addressed the problem of mass detection while disregarding the probable issue of class training imbalance caused by higher non-dense images in the training images that could contribute to lower Computer-Aided Diagnosis (CAD) establishment in clinical settings. In contrast, our study specifically brings the breast density into the focus of the learned parameter of the training images to adapt the class imbalance and improve the image before it can be trained to conduct mass detection, consequently bringing a good mass abnormality classifier. Nonetheless, limited studies have used metrics to compare their performance between non-dense and dense images before and after implementing their proposed work, making it difficult to make a suitable state-of-the-art analysis.

Although direct comparison is essentially incomparable between these works, both detection accuracy rate and testing time indicate that we achieved a better overall performance, which plays a significant role in showing that the proposed SbBDEM technique indeed increases the density-based performance. Our method outperformed works by [24,25] in terms of accuracy for non-dense and dense images. However, their work uses different datasets for a fair comparison. To the best of our knowledge, no study has been conducted using specifically the INbreast dataset with the metrics included for density-based mass detection. Meanwhile, work by [18] achieves 99.91% accuracy for benign and malignant classification compared to our method at 96% accuracy based on different breast densities. However, since the study’s augmentation process brings almost 7000 images from, originally, 112 images in INbreast, their work may cause unreliable results if the same technique is applied to a newer dataset. In contrast, the work of [45] exceeded our detection results for the same dataset. Nevertheless, it required more testing time than our approach due to the simpler training architecture employed. Additionally, since most of the studies listed applied CLAHE in their pre-processing stage, given that our enhancement method improves the detection model by mAP of 13.33% for CLAHE compared to the proposed SbBDEM technique as discussed in the result section, it is also expected to increase these studies detection stage if our pre-processing method is applied beforehand. Indeed, low accuracy limitations could be overcome by applying a more complex algorithm with more sophisticated hardware for training, which is expected to further improve the currently proposed SbBDEM technique for mass detection.

## 5. Conclusions

This work presents an image enhancement method according to the breast density level for Computer-Aided Diagnosis (CAD) stages for mammogram image analysis. Based on the result, the proposed SbBDEM technique could increase the performance for all stages of mass detection, segmentation, and classification for mammogram images. An improvement is observed when the proposed SbBDEM method is compared to the original image and the most widely used enhancement technique, i.e., contrast-limited adaptive histogram equalization (CLAHE) and histogram equalization (HE). The adjustment of the lower limit cap acts as a threshold value to separate the dense and mass to non-dense regions. This helps refine the textural information as a feature that represents both regions and through textural feature extraction in the classification stage, boosting the accuracy to 96% for the 5-fold cross-validation of benign vs. malignant classification experiment. The result also presents an improvement of mass detection with mean Average Precision (mAP) = 0.6401 to mAP = 0.8125, with mass detection in non-dense and dense accuracy of 93.33% and 95.33%, respectively. We achieved an increase of 98.41% confidence scores (CS) as opposed to 91.84% in the original image and a slight improvement of 0.03% in the mass segmentation using the proposed SbBDEM technique.

Meanwhile, in its original documentation, You Only Look Once v3 (YOLOv3) specializes in detecting smaller objects with the implementation of the second detection head. We further utilize this by modifying the second detection head into receiving the textural features that were already enhanced in the pre-processing stage through our proposed SbBDEM technique by adding these features to the deeper learning layer that contains more semantic information of the same image to improve the feature discrimination.

Our proposed method is limited by the unavailability of standardized image quality metrics that can determine the best image for all training images based on textural elements while considering the need for thousands of images for deep-learning purposes. While a high-quality image might be good for measuring accuracy, it is unnecessarily true to measure its textural aspect. Although statistical information for textural analysis is available, more suitable metrics can be investigated for more reliable metrics that relate image quality and texture. Additionally, with a running GPU capability of only 6 GB, the study is limited by the unavailability of a more sophisticated computing facility to employ higher-functioned YOLO, such as versions 4, 5, 6, and 7 without affecting the performance by reducing the mini-batches. However, the implementation of YOLOv3 in this study is sufficient as a way to demonstrate the effectiveness of density-based enhancement on the dataset before training and was modified based on its simplicity, which only runs on 5 MB or 1.2 million learnable parameters. Future studies could be explored by using other breast mammogram datasets with validation from a trained radiologist to enable CAD implementation in the medical field. Finally, the result obtained was comparable to the state-of-the-art performance from other methods discussed and can work as a base model for future updates by employing a more complex model on another dataset as well.

## Figures and Tables

**Table 1:** Average Quality Tests and GLCM features on INbreast Images (N = 112) using Enhancement Techniques.

No	Enhancement Techniques	MSE	BRISQUE	Mean Intensity	GLCM Textural Features			
					Contrast	Correlation	Energy	Homogeneity
**1**	Original	N/A	43.5799	0.5914	0.0276	0.9957	0.3174	0.9876
**2**	HE/AHE	0.0214	42.4518	0.6584	0.0758	0.9901	0.2212	0.9640
**3**	CLAHE	**0.0066**	42.9427	0.3786	0.0856	0.9933	0.1709	0.9621
**4**	SbBDEM	0.1169	**42.3841**	0.2302	0.0399	**0.9752**	**0.4339**	0.9803

**Table 2:** mAP performance for mass detection before and after YOLOv3 modification using different image enhancement techniques.

Image Condition	Mean Average Precision (mAP) Using YOLOv3 (%)	
	Without Modification	With Modification
Original	64.01	68.42
CLAHE	67.92	74.96
HE/AHE	57.40	54.35
Proposed	78.33	**81.25**

**Table 3:** Result of semantic segmentation for mass using different image input settings.

No	Image Input	Mean Accuracy	Mean IoU	IoU	
				Mass	Background
1	Original	0.9438	**0.8921**	0.8873	0.8970
2	HE/AHE	0.9385	0.8830	0.8775	0.8885
3	CLAHE	0.9423	0.8891	0.8844	0.8938
4	SbBDEM	**0.9441**	0.8917	**0.8878**	**0.8984**

**Table 4:** Comparison of CAD for mammogram mass detection previous works.

No	Authors	Enhancement Technique	Dense	Non-dense	mAP @0.5 Threshold	Overall Detection Acc (%)	Classification Acc (%)	Segmentation Acc (%)	Detection Time per Test Image
1	[10]	CLAHE	ROC = 0.902	ROC = 0.984	-	-	-	-	-
2	[24]	CLAHE	Acc = 91.00%	Acc = 94.80%	-	-	-	-	-
3	[25]	HE/AHE	Acc = 84.08%	Acc = 88.69%	-	-	-	-	-
4	[18]	CLAHE	-	-	-	-	99.91	-	-
5	[45]	CLAHE	-	-	-	98.96	95.64	92.97	12.3 s
6	[28]	HE/AHE	-	-	-	97.27	95.32	-	71 fps
7	[66]	-	-	-	0.9420 ^1^ 0.8460 ^2^	89.50	-	-	0.009 s
8	This Study	Proposed- SbBDEM	Acc = 93.33%	Acc = 95.33%	0.8125	92.61	96.00	94.41	1.78 s

^1^ Benign, ^2^ Malignant, Acc = Accuracy, ROC = Area under ROC curve, fps = frame per second.

## Data Availability

Updated dataset of INbreast is acquired through personal communication via email with the original author for latest updates.
